# Point-of-Care Ultrasound in Early Identification of Tamponade: A Case Series

**DOI:** 10.7759/cureus.78823

**Published:** 2025-02-10

**Authors:** Marina Kovacevic, Jonah M Cooper, Rachel Krater

**Affiliations:** 1 Hospital Medicine, Endeavor Health, Evanston, USA; 2 Internal Medicine Residency Program, University of Chicago (NorthShore), Evanston, USA; 3 Hospital Medicine, University of Michigan Health System, Ann Arbor, USA

**Keywords:** cardiac tamponade, cardiac tamponade, cardiac tamponade patients, pericardial effusion, pocus echocardiogram, point of care ultrasound

## Abstract

Cardiac tamponade is a life-threatening condition resulting from fluid accumulation within the pericardial sac, leading to impaired cardiac filling and output. Signs described in the literature, such as Beck’s triad (hypotension, jugular venous distension (JVD), muffled heart sounds) or pulsus paradoxus can be absent especially in the early stages, particularly in non-trauma patients, making early diagnosis challenging. In this case series, we present three patients who developed tamponade with subtle symptoms, including shortness of breath and tachycardia, but without hypotension or other hallmark signs. Cardiac point-of-care ultrasound (POCUS) played a critical role in early detection, revealing pericardial effusion and right ventricular (RV) diastolic collapse. Rapid diagnosis facilitated timely pericardiocentesis, which resolved symptoms and prevented further decompensation. This series underscores the value of POCUS in detecting early-stage tamponade, where traditional clinical markers of hypotension, JVD, muffled heart sounds, and pulsus paradoxus may not be present. The main purpose of this article is to showcase how early use of cardiac POCUS can prompt cardiology consultation and appropriate management, improving patient outcomes.

## Introduction

Fluid accumulation within the pericardial sac can progressively compress the right heart chambers, impair diastolic filling, reduce cardiac output, and ultimately lead to cardiac tamponade [1]. Tamponade is a life-threatening condition that can cause obstructive or cardiogenic shock, or a combination of both, resulting in cardiac arrest, if not promptly recognized and treated. Despite its severity, tamponade often presents with a non-specific clinical picture, making early diagnosis challenging.

Beck’s triad (hypotension, jugular venous distension (JVD), and muffled heart sounds) is traditionally considered a hallmark of tamponade. However, it was originally described in surgical populations and is rarely observed in its entirety in clinical practice [2]. Another commonly cited finding, pulsus paradoxus (a decrease in systolic blood pressure by more than 10 mmHg with inspiration), may be absent in patients with elevated diastolic pressures, pulmonary hypertension, aortic regurgitation, or atrial septal defect [1,3-5]. In non-trauma patients, pericardial fluid accumulation often develops gradually, leading to a subtle presentation with non-specific symptoms such as shortness of breath, chest pressure, and tachycardia.

Electrocardiographic findings such as low voltage QRS complexes and electrical alternans (beat-to-beat variation in QRS amplitude) are frequently described but are relatively rare. More commonly, sinus tachycardia is the predominant electrocardiographic finding in tamponade. Chest X-ray may show an enlarged cardiac silhouette, though this is a non-specific finding. Additionally, early-stage tamponade may lack overt signs of shock [3-5]. Given that clinical signs and symptoms depend on the rate of fluid accumulation and pericardial compliance and that hallmark findings are often incomplete, tamponade remains a difficult clinical diagnosis.

Echocardiography is a gold standard for tamponade diagnosis but is limited by availability and operator dependency. In this context, cardiac point-of-care ultrasound (POCUS) has emerged as a valuable, accessible, and cost-effective tool for the early detection of cardiovascular emergencies, including tamponade [6]. Performed at the bedside by trained clinicians, cardiovascular POCUS is a problem-oriented imaging approach that is becoming an integral part of routine medical practice [7].

POCUS serves as an adjunct to the physical examination and has broad applications, from trauma assessment in the emergency department (ED) to evaluating patients in shock or during resuscitation. In inpatient settings, POCUS can guide diuresis in acute heart failure using protocols such as reverse fluid administration limited by lung sonography (FALLS) or cardiopulmonary limited ultrasound examination (CLUE), which also assist in assessing left ventricular function and left atrial size [8,9]. The venous excess ultrasound (VExUS) technique further allows trained users to evaluate volume status and venous congestion by assessing the inferior vena cava (IVC), hepatic, portal, and intrarenal veins [10]. Additionally, POCUS enhances the diagnostic accuracy of valvular heart disease, proving superior to cardiac auscultation [11].

The significance of POCUS in diagnosing pericardial effusion lies in its immediate availability and ability to promptly detect effusion while simultaneously assessing its hemodynamic impact [12]. It enables visualization of right atrial (RA) collapse and right ventricular (RV) diastolic collapse, both indicative of elevated intrapericardial pressure due to tamponade physiology. Furthermore, it allows for the assessment of IVC size and respiratory variation, indirectly estimating RA pressure.

Here, we present three cases demonstrating the early detection of cardiac tamponade in hemodynamically stable patients using cardiac POCUS. We propose that trained POCUS users can facilitate the early identification of tamponade, potentially bridging the gap in traditional diagnostic strategies that rely solely on formal echocardiography. This may prevent clinical deterioration and enable timely intervention in patients with tamponade, presenting with non-specific symptoms.

## Case presentation

Case 1

**Figure 1:**
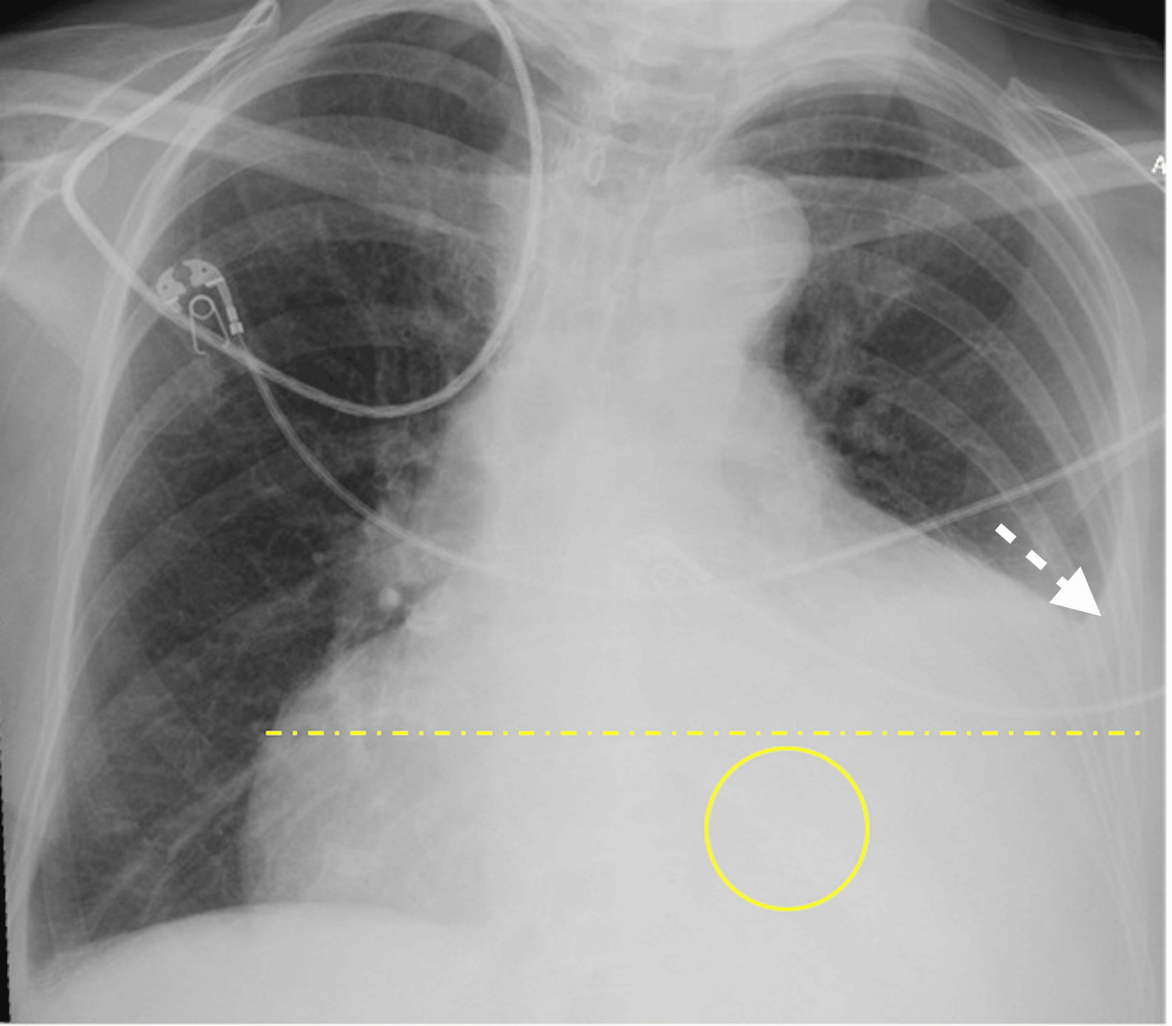
Chest X-ray, AP upright portable view Marked cardiomegaly (dashed yellow line). Moderate  left pleural effusion (white arrow). Retrocardiac opacity (circle). AP: Anteroposterior

An 84-year-old female presented to the ED with worsening shortness of breath, malaise, and a dry cough. Her medical history was significant for heart failure with preserved ejection fraction (HFpEF) (EF 62%, no prior pericardial effusion), non-valvular atrial fibrillation on rivaroxaban, chronic lymphocytic leukemia (CLL) with left cervical and right axillary lymph node involvement, chronic left pleural effusion attributed to HFpEF (no prior thoracentesis), and stage 3 chronic kidney disease (CKD3). On presentation, she had normal vital signs except for saturation of 86% oxygen on room air, requiring 2 L/min of oxygen supplementation through nasal cannula. Her electrocardiogram (ECG) was notable for atrial fibrillation with normal rate. Initial chest X-ray showed slightly increased size of left pleural effusion, trace right pleural effusion, cardiomegaly and a retrocardiac consolidation (Figure 1).

Pertinent laboratory results included white blood cell count (WBC) 13.4×10³/µL (reference range 4-10×10³/µL), blood urea nitrogen (BUN) 64 mg/dL (reference range 8-23 mg/dL), and creatinine (Cr) 2.7mg/dL (baseline 1.4 mg/dL, reference range 0.5-0.9mg/dL). Physical examination was notable for pitting edema extending to the knees. As the patient had been holding torsemide during recent vacation a week prior, she was preliminarily diagnosed with acute on chronic HFpEF exacerbation and overlying pneumonia (based on the read of retrocardiac opacity on the chest X-ray).

**Figure 2:**
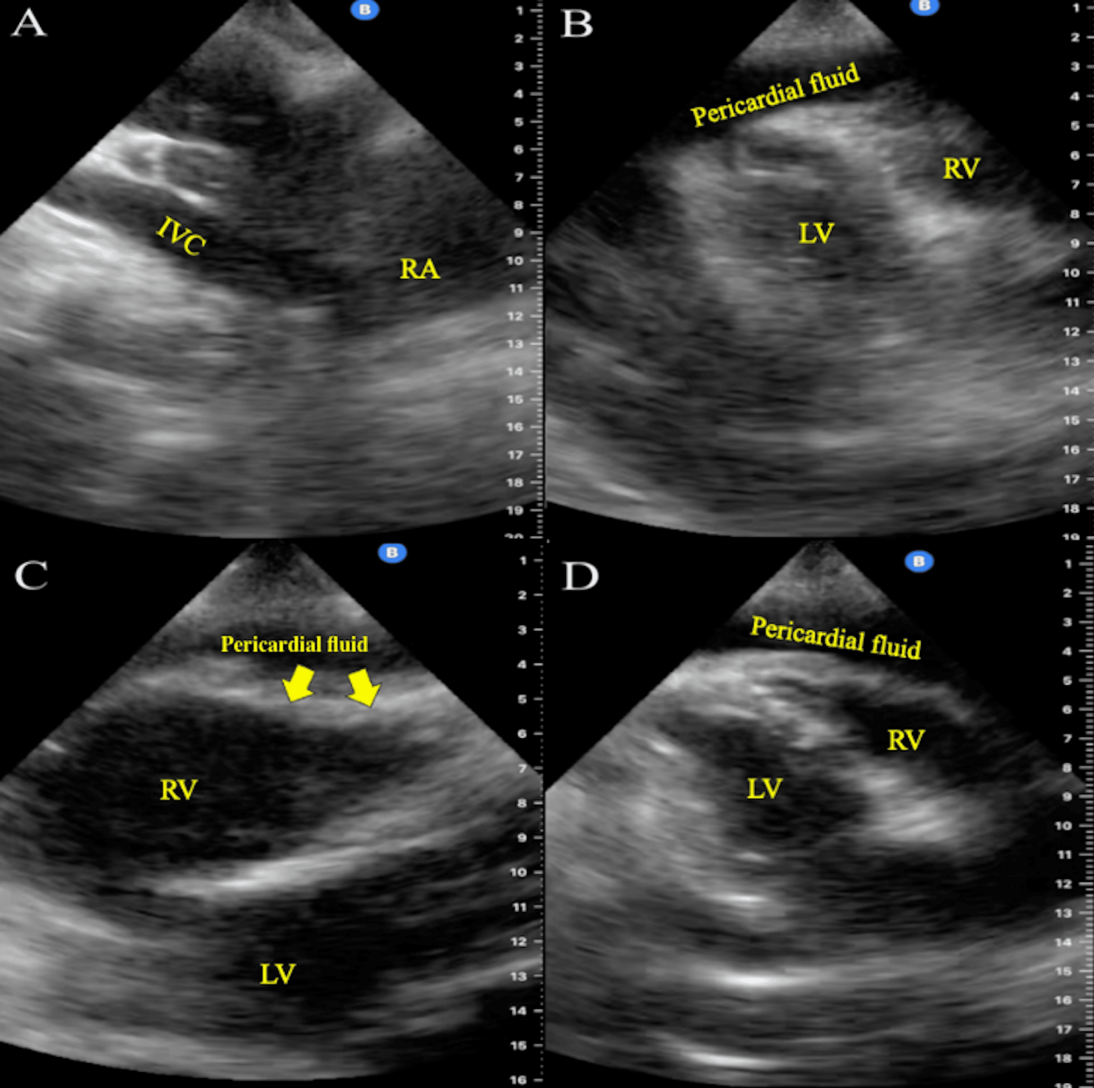
Case 1 POCUS images showing large pericardial effusion compressing right ventricle A: Subxiphoid view. Dilated IVC entering the right atrium. IVC diameter appears > 2.1 cm. B: Parasternal long axis view. Moderate-to-large pericardial effusion (pericardial fluid) ~1.5-2 cm in diameter is visualized expanding along the apical myocardium. C: Subxiphoid view. RV bowing (indicated by yellow arrows) secondary to pericardial effusion (pericardial fluid). Demonstrative of RV diastolic collapse. D: Parasternal long axis view. RV collapse secondary to effusion (pericardial fluid). POCUS: Point-of-care ultrasound; IVC: Inferior vena cava; RV: Right ventricular

**Figure 3:**
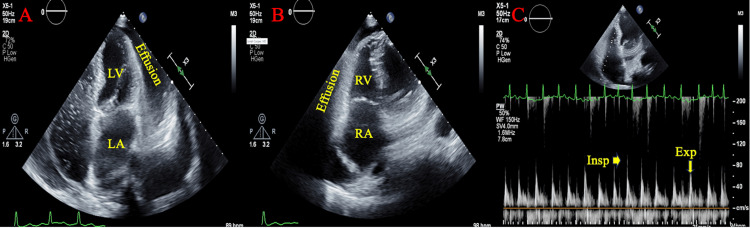
A) Four-chamber view demonstrating presence of Effusion. Left atrium appears dilated; B) Four-chamber view, focused on right side of the heart, Effusion is redemonstrated alongside the right ventricle, right atrium is dilated; C) Excessive respiratory variation over tricuspid valve demonstrated through both the insp and exp phases of respiration Effusion: Large circumferential pericardial effusion; insp: Inspiratory; exp: Expiratory

After provisional treatment with ceftriaxone, azithromycin, and 20 mg of IV furosemide, she was admitted to the medical unit. After admission, two additional IV furosemide 20 mg doses were provided with minimal urinary response. Overnight, she developed worsening right-sided chest tightness and shortness of breath. No specific interventions were provided at this time. The following morning, the patient was additionally noted to have upper airway wheezing and had no urinary response to furosemide. Vitals had remained unchanged and stable. Repeat laboratory work revealed WBC 15×10³/µL, BUN 71 mg/dL, and Cr 2.9 mg/dL. Given the ambiguous presentation, lack of response to treatment, and the availability of a POCUS-trained physician, a handheld POCUS device was used as an adjunct to the standard physical examination for a more comprehensive assessment of the patient. In addition to the left pleural effusion, POCUS revealed a new moderate-to-large pericardial effusion with RV bowing on parasternal long-axis and subxiphoid views, along with a dilated IVC, raising concerns for cardiac tamponade (Figure 2). Vital signs remained normal. Antibiotics and diuresis were stopped. Urgent cardiologic consultation was obtained, along with a formal echocardiogram which confirmed tamponade (Figure 3).

The patient underwent a same-day pericardiocentesis via subxiphoid approach and a subsequent pericardial drain placement. The intervention provided relief of all symptoms, including resolution of wheezing and improvement of acute kidney injury. Fluid analysis was unremarkable, with no growth on cultures and unremarkable cytology, ruling out bacterial or malignant pericardial effusion. Following successful treatment of acute pericarditis, the patient was discharged on hospital day four after removal of the pericardial drain, with a final diagnosis of likely viral pericarditis. She was advised to continue colchicine treatment and to further follow-up with cardiology.

Case 2

**Figure 4:**
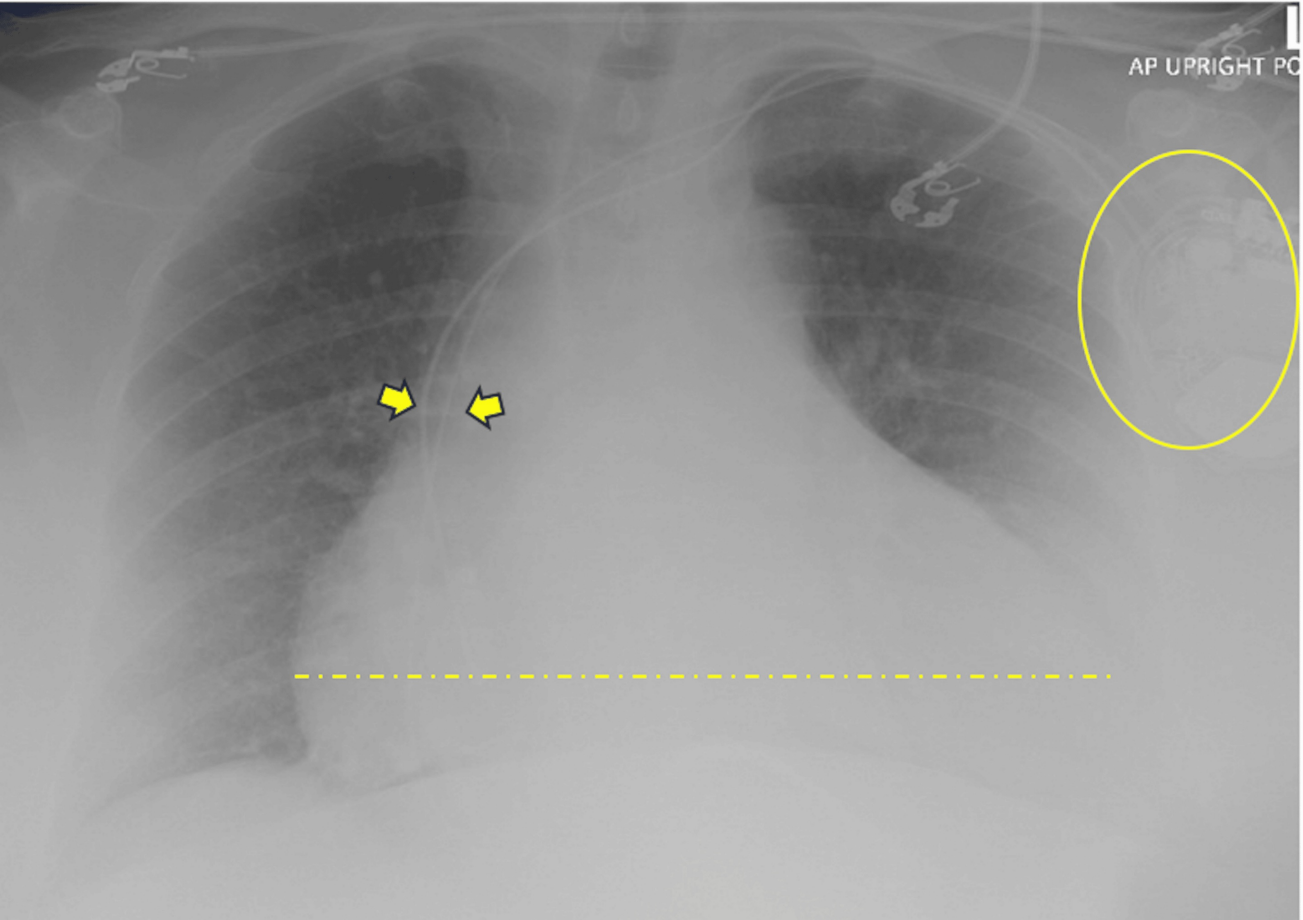
Chest X-ray, AP upright portable view Marked cardiomegaly (dashed line) without evidence of pulmonary edema or consolidation. Dual-lead left-sided cardiac defibrillator (circled) is demonstrated with stable position of leads (arrows). AP: Anteroposterior

A 75-year-old female presented to the ED for worsening shortness of breath. Past medical history included apical hypertrophic obstructive cardiomyopathy (HOCM), a history of ventricular tachycardia (VT) with an automatic implantable defibrillator (AICD), HFpEF (EF 77%, no previous history of pericardial effusion), non-valvular atrial fibrillation (on apixaban), and hypertension. On presentation, she was noted to be in atrial fibrillation on ECG with a heart rate of 125 beats per minute (bpm) but otherwise had normal vital signs. Her laboratory work was significant for WBC 12.9×10³/µL and Cr 1.9 mg/dL (baseline 1.0 mg/dL). Chest X-ray demonstrated a markedly enlarged heart and no significant pulmonary findings (Figure 4). Atrial fibrillation was treated with diltiazem drip and the patient was transferred to the general medical floor.

**Figure 5:**
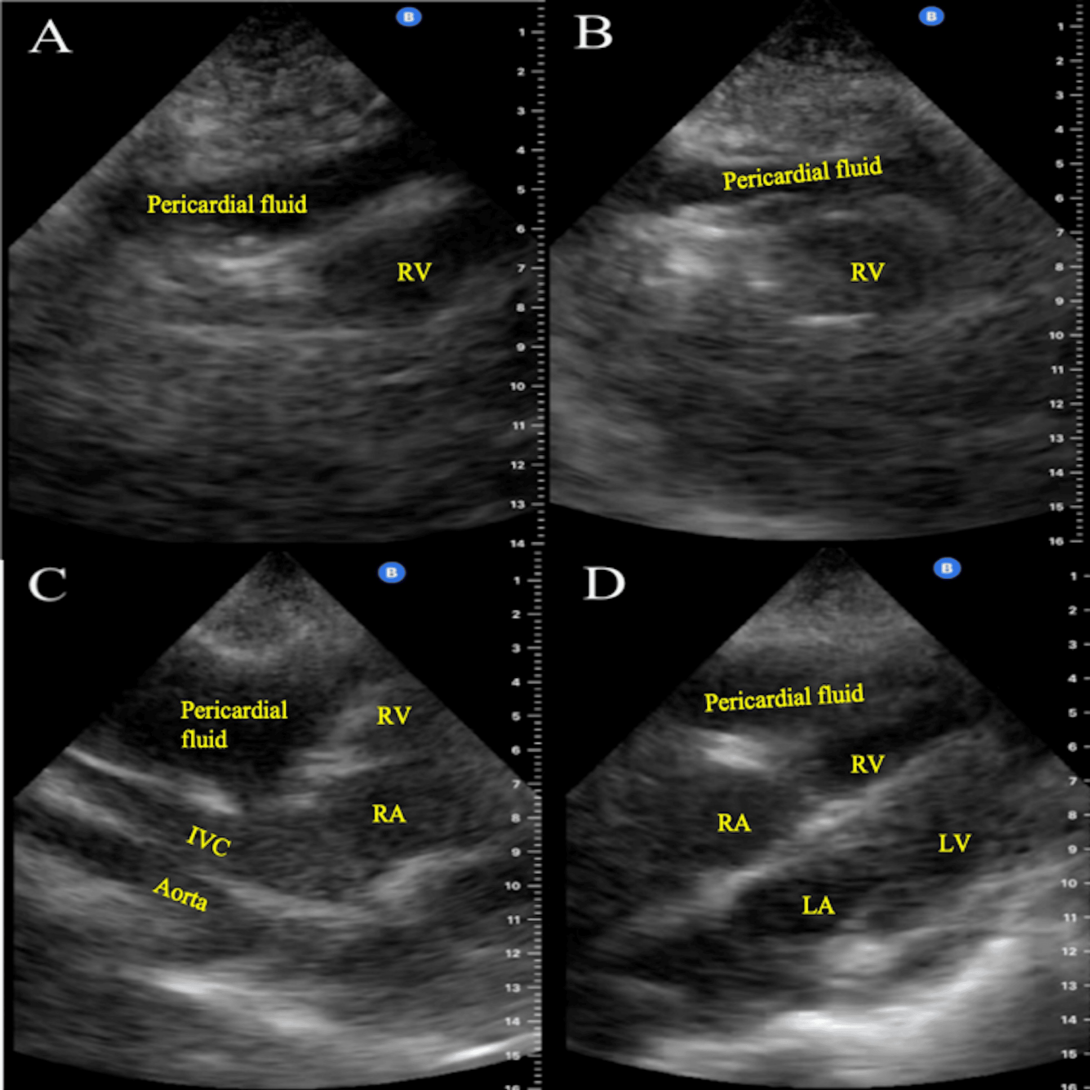
Case 2 (Images A and B) and case 3 (Images C and D) showcasing pathology concerning for tamponade A: Case 2, Subxiphoid view. RV diastolic collapse secondary to, in this view, large (~2 cm) pericardial effusion. B: Case 2, Subxiphoid view. Moderate amount fluid (~1.2 cm) from pericardial effusion can be seen surrounding the surface of the right ventricle. C: Case 3, Subxiphoid view. IVC entering the right atrium. IVC diameter appears ~2cm. Large pericardial effusion (>2cm) can be seen. D: Case 3, Subxiphoid view. Large pericardial effusion (>2cm)  can be seen pressing against the right atrium and right ventricle. RV: Right ventricular; IVC: Inferior vena cava

Based on the chest X-ray findings of an enlarged cardiac silhouette, medical team raised concern for a possible pericardial effusion. With the availability of a POCUS-trained physician, a bedside POCUS examination using a handheld device was performed, confirming the presence of pericardial effusion. In the subxiphoid view, POCUS also revealed RV bowing and diastolic collapse, raising concern for cardiac tamponade (Figure 5A, 5B). IVC was not visualized. At the time of POCUS examination, all vital signs were normal other than the patient’s atrial fibrillation, with a rate ranging from 104 to 113 bpm on diltiazem drip.

**Figure 6:**
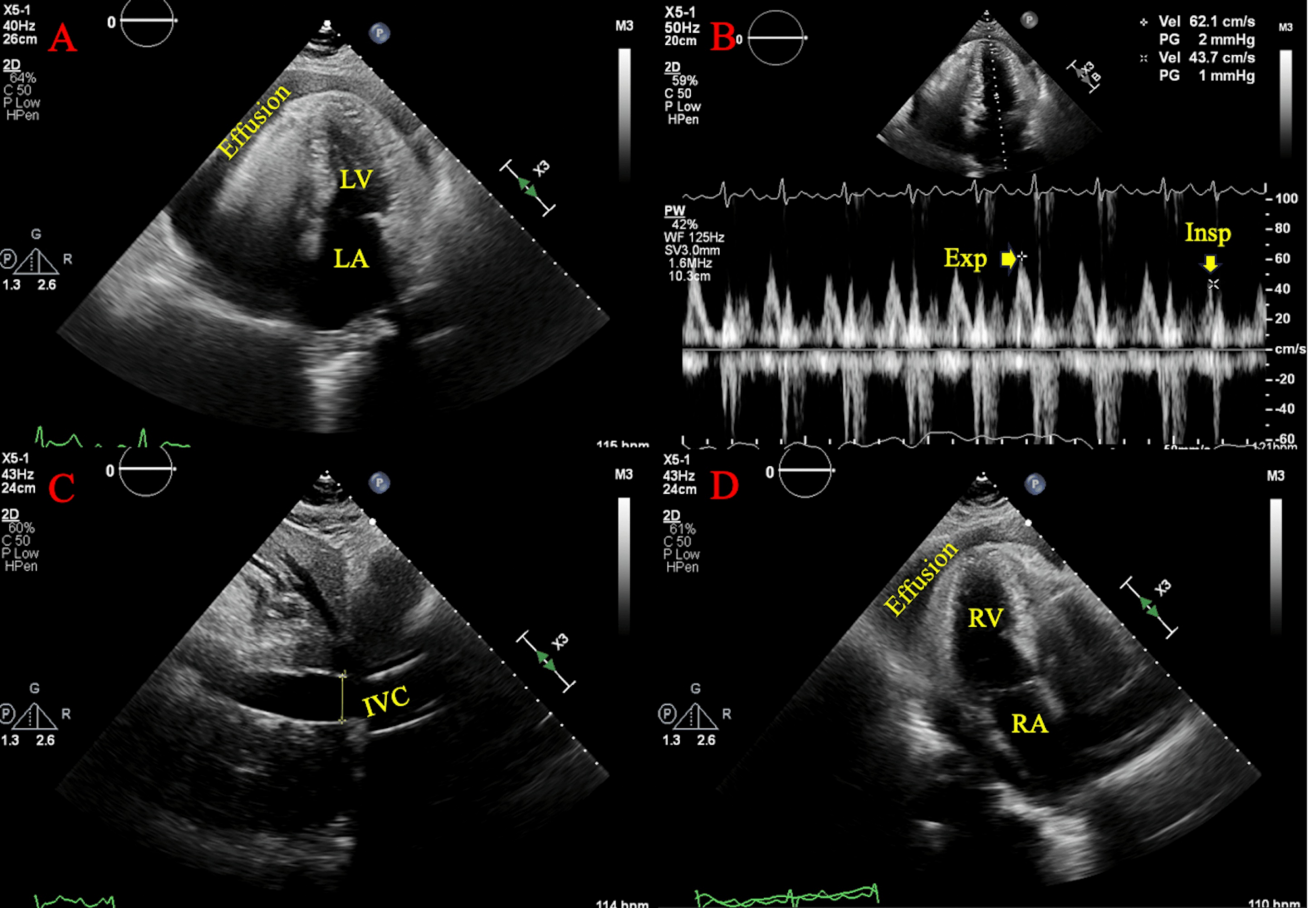
A) Four-chamber view, large pericardial effusion is visualized. Moderately thickened left ventricle apex and septum. Left atrium and left ventricle noted to be compressed by effusion; B) Excessive respiration variation across the mitral valve noted with insp and exp indicated; C) IVC was dilated with a diameter of 2.4 cm and less than 50% respiratory variation; D) Effusion is redemonstrated surrounding the right ventricle. Right atrium appears inverted insp: Inspiratory; exp: Expiratory; IVC: Inferior vena cava

Cardiology was consulted, a formal echocardiogram was obtained and apixaban was held for possible pericardiocentesis pending results. Echocardiogram confirmed pericardial effusion with tamponade physiology based on RV impingement, IVC with decreased collapsibility index and increased respiratory variation over the tricuspid and mitral valves (Figure 6). Due to recent apixaban usage, and in a setting of normal vital signs, pericardiocentesis was delayed. By the following day, the patient had developed chest pain, was hypotensive, and developed worsening acute kidney injury (Cr was 3.7 mg/dL with hyperkalemia of 5.4 mEq/L, reference range for potassium level being 3.5-5.3 mEq/L). Pericardiocentesis was immediately performed under ultrasound guidance, 850 mL of hemorrhagic fluid was drained with resolution of the effusion and a pericardial drain was placed. The patient’s symptoms and vital signs stabilized. Pericardial fluid analysis, including cytology and cultures, was unremarkable and the patient was diagnosed with idiopathic pericarditis in the setting of oral anticoagulation use. The pericardial drain was removed on the fourth day of hospitalization and apixaban was resumed the following day. The patient was started on ibuprofen and colchicine to reduce pericardial inflammation and discharged from the hospital on the fifth day of hospitalization with cardiology follow-up. 

Case 3 

**Figure 7:**
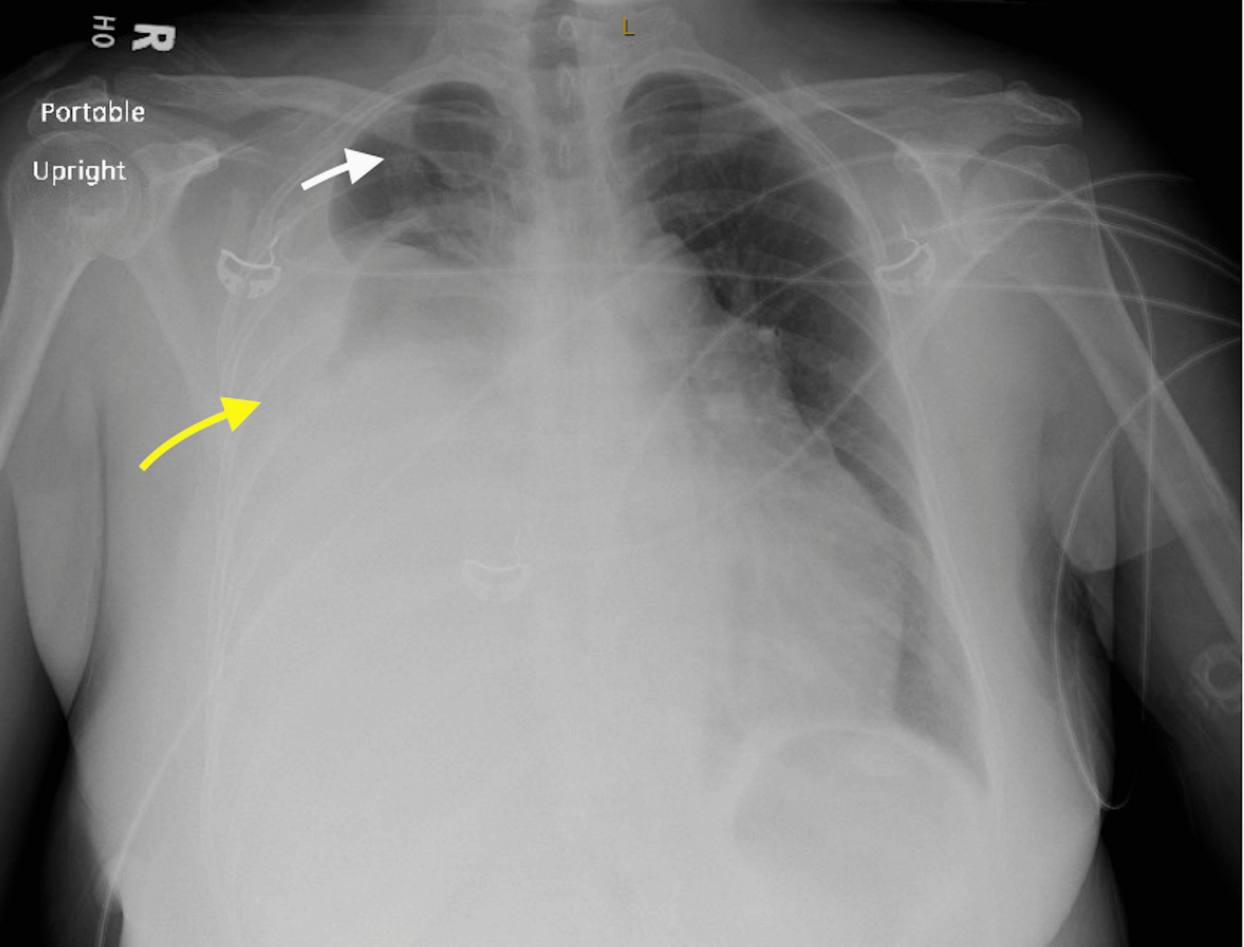
Chest X-ray, AP upright portable view Opacification of most of the right hemithorax due to large right pleural effusion (yellow arrow) with small area of remaining aeration in the apex (white arrow). Right perihilar mass is obscured. AP: Anteroposterior

A 55-year-old female presented to the ED from the oncology clinic after she was noted to have significant shortness of breath. Past medical history included recently diagnosed anterior mediastinal stage four biphasic cancer (with mesenchymal component and rhabdomyoblastic differentiation), with metastases to the pleura and right twelfth rib, malignant right pleural effusion, and no previous history of pericardial effusion. Despite multiple thoracenteses during the month prior, the patient noticed continual worsening of her shortness of breath and dry cough. In the ED, the patient had normal vital signs except for tachycardia, tachypnea (40 breaths per minute), saturating 86% on room air and requiring 2 L/min of oxygen supplementation through nasal cannula. Her ECG was notable for atrial fibrillation with a rapid ventricular response (RVR) of 133 bpm. The patient’s laboratory work was only significant for hyponatremia of 123 meq/L (reference range 133-145 meq/L). Initial chest X-ray was notable for near complete opacification of the right lung with evidence of a pleural effusion (Figure 7).

The patient was started on a diltiazem drip and transferred to the ICU for non-invasive positive pressure ventilation (NIPPV). On arrival to the ICU, the patient underwent right-sided thoracentesis with partial improvement of her symptoms and normalization of the heart and breathing rate. She was weaned off NIPPV overnight and transferred to the general medical floor in the morning. One day after, she experienced worsening shortness of breath, increasing work of breathing, and recurrence of atrial fibrillation with RVR. Laboratory work was unremarkable, including sodium.

**Figure 8:**
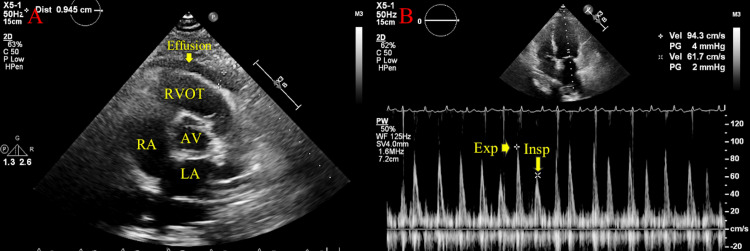
A) Large, circumferential pericardial effusion along the RV free wall, measured 2.5 cm anteriorly. RVOT, AV, right atrium, and left atrium are labelled; B) Increased respiratory variation noted (>30%) across the mitral valve, suggestive of tamponade physiology RV: Right ventricular;  RVOT: Right ventricular outflow tract; AV: Aortic valve

Bedside POCUS examination via handheld device was used to assist physical examination. In subxiphoid view, it showed pericardial effusion with associated RV diastolic collapse raising concern for cardiac tamponade (Figure 5C, 5D). Visualized IVC had less than 50% respiratory variations and was around 2 cm in diameter, suggesting increased RA pressure (IVC with 50% respiratory variations and diameter <2.1 cm favors normal RA pressure). At the time of POCUS, vital signs were normal, including blood pressure and pulse, with saturation of 97% on 2 L nasal cannula. Cardiology was consulted, and due to the absence of JVD and pulsus paradoxus on physical examination, there was little concern for actual tamponade. However, a same-day formal echocardiogram raised concern for early hemodynamic compromise due to presence of large pericardial effusion associated with increased retrophasic variation across the mitral valve (>30% variation), which is alternative to pulsus pradoxus (Figure 8). 

Pericardiocentesis was pursued the same day, with removal of 150 mL of serosanguinous pericardial fluid as well as pericardial drain placement. Initial pericardial pressure was notably 20 mmHg and final pericardial pressure was 14 mmHg (normal range from -5 to 5 mmHg), additionally confirming tamponade. Patient was diagnosed with malignant pericardial effusion, with cytology being consistent with her previous cancer history. As of the following day, the patient reported symptomatic improvement of her shortness of breath, oxygen requirement decreased, and her heart rate returned within normal limits. Prior to discharge, the patient had pericardial drain removed and an indwelling pleural catheter placed. Symptoms remained quiescent thereafter.

## Discussion

Diagnosing cardiac tamponade begins with recognizing its most pertinent symptoms and physical examination findings. The most commonly reported symptom is shortness of breath, occurring in 66-90% of cases, followed by chest pain in 12-74% of patients. On physical examination, tachycardia (75% sensitivity) and tachypnea (80% sensitivity) are the most frequently observed findings, though both are non-specific. JVD, often considered a classic sign, has a low sensitivity of only 12.5% [4]. Pulsus paradoxus, though a valuable diagnostic tool with a sensitivity of 98% and specificity of 83%, is underutilized, with only 35% of patients with tamponade recognized to have this finding during bedside assessment [13]. The widely referenced Beck’s triad is relatively rare, with a retrospective study by Stoltz et al. reporting its sensitivity as 0-19.4% in patients with tamponade [3]. Hypotension, a late sign indicative of progressing shock, is often absent in the early stages of tamponade [5]. 

While tamponade is a clinical diagnosis, echocardiography remains the gold standard for confirmation. Other supporting tests, such as ECG and chest X-ray, offer additional but often unreliable clues. ECG findings vary, with low QRS voltage demonstrating a sensitivity of 69% in chest leads and 67% in limb leads. Electrical alternans, though highly specific (100%), has a sensitivity of only 5% [14]. Chest X-ray may reveal an enlarged cardiac silhouette, but has a sensitivity of 71% and a low specificity of 41% [15].

Diagnosis of tamponade by echocardiogram is based on the presence of RA and RV collapse, a plethoric IVC with reduced respiratory variation, and exaggerated respiratory inflow variations across the mitral and tricuspid valves. RA systolic collapse is the earliest echocardiographic sign of tamponade, occurring when intrapericardial pressure exceeds RA pressure. RA collapse lasting more than one-third of systole is highly sensitive (94%) and specific (100%) [16,17]. In late stages of tamponade, RA diastolic collapse can also be seen (with both sensitivity and specificity nearing 100%). RV diastolic collapse, initially observed during inspiration, has a sensitivity of 60-90% and specificity of 95-100% [16,17]. IVC plethora (diameter >2.1 cm with <50% respiratory variation) suggests elevated RA pressure and is highly sensitive (95-97%) but not specific (approximately 40%) [12,18]. Mitral and tricuspid flow variabilities are important echocardiographic findings. Mitral inflow velocity typically decreases slightly with inspiration (less than 25%), while tricuspid inflow velocity decreases more noticeably with expiration (less than 40%) due to the changes in intrathoracic pressure during the respiratory cycle. In tamponade, due to increased intrapericardial pressure, an inspiratory mitral inflow decrease more than 25% and a tricuspid inflow increase more than 40% are diagnostic of tamponade and serve as echocardiographic surrogates for pulsus paradoxus [19].

In our case series, all three patients presented with non-specific symptoms, including shortness of breath and chest discomfort. Physical examination revealed tachycardia and atrial fibrillation, but no JVD or pulsus paradoxus were noted or looked for. Vital signs remained stable without significant hypotension. None exhibited electrical alternans or low QRS voltage on ECG, and chest X-ray findings were limited to an enlarged cardiac silhouette.

Cardiac POCUS was performed by three different physicians, each with one to three years of POCUS experience and training from at least two national POCUS conferences. They pursued POCUS due to uncertainty in clinical presentation and inadequate treatment response, identifying pericardial effusions in all three cases. Notably, normal vital signs were reported as a misleading factor, initially challenging the diagnosis of tamponade despite the findings on POCUS.

POCUS has multiple utilities in assessing pericardial fluid, starting by assessing its size and characteristics (anechoic, echogenic and complex fluid; indicating potential etiology). The size of effusion is measured at the widest point between two pericardial layers in diastole in parasternal long axis. Small effusions (<10 mm) correspond to approximately 300 mL of fluid, moderate effusions (10-20 mm) to around 500 mL, and large effusions (>20 mm) to approximately 700 mL [12]. After identifying pericardial fluid, its hemodynamic impact should be assessed through visualization of RA collapse and RV diastolic collapse (both indicative of increased intrapericardial pressure) [12,19]. IVC should be evaluated next by assessing its size and respiratory variations. These help approximate the RA pressure. Normal RA pressure of 0-5 mmHg is suggested by IVC size <2.1 cm and >50% of IVC collapse during expiration ("sniff test"), while IVC >2.1 cm and reduced respiratory variability <50% indicate increased RA pressure (10-20 mmHg) [20]. Additional POCUS finding suggestive of tamponade is abnormal movement of interventricular septum (septal bounce). Changes in inflow velocities over mitral and tricuspid can also be estimated by use of doppler mode in cardiac POCUS, however are limited to more advanced users [12]. 

The accuracy of POCUS in detecting pericardial effusion is reported to be 97.6%, even among physicians without advanced training [21]. Beyond identifying the effusion, POCUS enables bedside evaluation of RA collapse, RV diastolic collapse, and IVC assessment, providing real-time insights into a patient’s hemodynamics and tamponade diagnosis. This, in turn, allows for expedited triaging and prompt cardiac consultation when necessary. Literature suggests that the time to pericardiocentesis can be significantly reduced in patients who undergo POCUS in the ED, with a median time of 11.3 hours in the POCUS arm compared to 70.2 hours for those patients whose diagnosis relied solely on departmental echocardiography [22]. POCUS facilitates faster diagnostic turnaround compared to traditional imaging pathways.

When using POCUS, especially with handheld devices, certain cardiac structures can be difficult to visualize, especially if user is inexperienced [7]. In our cases, we encountered challenges in visualizing the right atrium, and assessing for RA systolic collapse, which is typically the earliest sign of tamponade. Additionally, visualizing and assessing the size of IVC can also be challenging. Despite these limitations, we were able to easily detect pericardial effusions associated with RV collapse, raising concern for development of tamponade. Further development of artificial intelligence (AI)-assisted POCUS applications can help in image acquisition and interpretation and in improving quality assurance and control.

In summary, symptoms, physical examination findings, ECG, and chest X-ray suffer from either low sensitivity or lack specificity when diagnosing tamponade, making the tamponade diagnosis challenging. This is especially the case in its early stages, when timely treatment could prevent clinical deterioration. Although often emphasized in the literature as hallmarks of tamponade, hypotension and signs of cardiovascular collapse actually signal advanced tamponade. Relying solely on signs of shock, Beck’s triad, and pulsus paradoxus to diagnose tamponade can delay the actual diagnosis and timely treatment. This is where the role of cardiac POCUS is highlighted as an important adjunct to physical examination, especially when there is no clear indication for pursuing formal echocardiogram and when there is ambiguity in clinical presentations and treatment responses. In the three cases we reported, POCUS prompted early diagnosis of cardiac tamponade prior to development of cardiovascular compromise, expediting cardiology consultation, formal echocardiography, and reducing time to pericardiocentesis.

While POCUS should not replace a formal echocardiogram for diagnosing cardiac tamponade, it serves as a valuable screening tool to raise concern for this condition. As POCUS becomes increasingly integrated into clinical practice, it allows clinicians to more promptly investigate potential life-threatening emergencies like cardiac tamponade based on clinical suspicion, such as shortness of breath, chest pressure, tachycardia, or an enlarged cardiac silhouette on chest X-ray, especially in at-risk patients (e.g., those with active cancer, connective tissue disorders, or renal failure), rather than waiting for advanced imaging or further clinical deterioration. With adequate training, clinicians can use POCUS to identify tamponade, initiate emergent treatment, or refer stable patients with high clinical suspicion for a formal transthoracic echocardiogram. 

## Conclusions

In conclusion, our cases emphasize the crucial role of cardiac POCUS in the early diagnosis and treatment of cardiac tamponade. Common symptoms like shortness of breath and chest pain are non-specific, while clinical findings often described in literature, like Beck's triad or pulsus paradoxus, are not frequently noted in tamponade patients. The non-specific nature of ECG and chest X-ray findings further underscores the limitations of conventional diagnostic methods, and formal echocardiography may not be pursued due to non-specific clinical picture or may not be readily available. Regular utilization of POCUS can lead to timely detection and intervention in patients with cardiac tamponade. This can be valuable in POCUS users of various experience, and particularly with more experienced POCUS users able to measure transmitral flow variations. Given its potential to enhance clinical assessment when diagnosis is uncertain, POCUS should be incorporated more liberally into routine practice as an important adjunct to physical examination, further improving patients' outcomes.
